# Tumor necrosis factor and norepinephrine lower the levels of human neutrophil peptides 1-3 secretion by mixed synovial tissue cultures in osteoarthritis and rheumatoid arthritis

**DOI:** 10.1186/ar3044

**Published:** 2010-06-04

**Authors:** Birgit Riepl, Susanne Grässel, Reiner Wiest, Martin Fleck, Rainer H Straub

**Affiliations:** 1 Laboratory of Experimental Rheumatology and Neuroendocrino-Immunology, Division of Rheumatology, Department of Internal Medicine I, University Hospital, F.J. Strauss Allee 11, 93053 Regensburg, Germany; 2 Department of Orthopedic Surgery, University Hospital Regensburg, Asklepios Clinic Bad Abbach, Kaiser-Karl-V.-Allee 3, 93077 Bad Abbach, Germany

## Abstract

**Introduction:**

Neutrophils and monocytes play an important role in overt inflammation in chronic inflammatory joint diseases such as rheumatoid arthritis (RA). The sympathetic nervous system (SNS) inhibits many neutrophil/monocyte functions and macrophage tumor necrosis factor (TNF), but because of the loss of sympathetic nerve fibers in inflamed tissue, sympathetic control is attenuated. In this study, we focused on noradrenergic and TNF regulation of human neutrophil peptides 1-3 (HNP1-3), which are proinflammatory bactericidal α-defensins.

**Methods:**

Synovial tissue and cells were obtained from patients with RA and osteoarthritis (OA). By using immunohistochemistry and immunofluorescence, HNP1-3 were tracked in the tissue. With synovial cell-culture experiments and ELISA, effects of norepinephrine, TNF, and cortisol on HNP1-3 were detected.

**Results:**

HNP1-3 were abundantly expressed in the synovial lining and adjacent sublining area but not in deeper layers of synovial tissue. The human β-defensin-2, used as control, was hardly detectable in the tissue and in supernatants. HNP1-3 double-stained with neutrophils but not with macrophages, fibroblasts, T/B lymphocytes, and mast cells. Norepinephrine dose-dependently decreased HNP1-3 levels from RA and OA cells. TNF also inhibited HNP1-3 levels from OA but not from RA cells. Cortisol inhibited HNP1-3 levels only in OA patients. A combination of norepinephrine and cortisol did not show additive or synergistic effects.

**Conclusions:**

This study demonstrated an inhibitory effect of norepinephrine on HNP1-3 of mixed synovial cells. In light of these findings, the loss of sympathetic nerve fibers with low resting norepinephrine levels might also augment the inflammatory process through HNP1-3.

## Introduction

Rheumatoid arthritis (RA) is a chronic joint disease leading to severe erosions of adjacent bone, which are not observed in patients with osteoarthritis (OA). Although an inflammatory process is present in OA synovial tissue, RA patients demonstrate a higher state of synovial tissue inflammation compared with OA patients. In the pathophysiology of RA, T cells, B cells, macrophages, fibroblasts, and osteoclasts play dominant roles. In addition, neutrophils are important mediators of tissue inflammation in RA, and neutrophils are the most abundant cell type in the synovial fluid [1]. Neutrophil production of proteases, reactive oxygen species, S100 proteins, cytokines, chemokines, and complement stimulate inflammation [2]. Fc-gamma receptors on neutrophils can bind immune complexes that can perpetuate the inflammatory process [2]. In addition, neutrophils produce important antimicrobial proteins, such as defensins [3].

A large number of defensins and defensin-like peptides have been reported in many organisms. As of March 2010, 363 entries had been recorded in a defensin database [4]. One distinguishes α-defensins (in neutrophils) from β-defensins (in other cells). Human neutrophils contain four α-defensins (HNP-1 to HNP-4) [5]. HNPs are unique to neutrophils and account for ~99% of the total α defensin content of these cells [5]. HNPs exert chemotactic, immunomodulating, and cytotoxic effects and participate in inflammation [5]. In contrast, human β-defensins have been described mainly in epithelial cells but also in leukocytes, heart, skeletal muscle, testis, keratinocytes, tonsil, placenta, and other tissues [4]. Defensins are expressed and released on bacterial stimuli involving the Toll-like receptors and, in addition, other stimuli, such as cytokines [6]. Besides microbicidal activities, defensins can stimulate TNF secretion from macrophages, as recently reported [7].

In tissues of patients with RA and OA, the investigation of defensins has recently begun. One report demonstrated the presence of HNP1 3 positive cells in synovial tissue of healthy subjects and in patients with suppurative arthritis, osteoarthritis (OA), and RA [8]. However, regulation of HNP1-3 in these diseases (for example, by cytokines) has not yet been investigated. Another report found an association of HNP1-3 in synovial fluid of patients with RA and severe erosive joint disease [9]. These studies clearly demonstrate that neutrophil defensins are present in inflamed tissues of patients with RA and OA. Some reports also demonstrated that defensins can be produced by dendritic cells and monocytes [10,11], but this has not been demonstrated in synovial tissue or cells of RA and OA patients.

For several years, we have been interested in the role of the sympathetic nervous system (SNS) in OA, RA, and experimental arthritis [12,13]. We have been attracted by effects of the SNS on neutrophils and monocytes, but effects on defensin secretion are presently not known. It is recognized that activation of the SNS enhances neutrophil and monocyte mobilization, leading to increased numbers of circulating neutrophils and monocytes, called the first line of defense [14-16]. Such a mechanism might increase the numbers of neutrophils and monocytes that enter inflamed tissue at sites of leaky endothelial structures (leakiness is important because catecholamines inhibit neutrophil and monocyte attachment to normal endothelium). However, once neutrophils and monocytes have entered inflamed tissue, the major neurotransmitter of sympathetic nerve fibers, norepinephrine, inhibits several neutrophil and monocyte functions. For example, norepinephrine decreases migration [17,18], oxygen radical production [19], phagocytosis [18], and bactericidal activity [20]. These inhibitory influences were present only when norepinephrine appeared at high concentrations (via β_2_-adrenergic receptors). This might be quite different at low concentrations when norepinephrine exerts its effects via α-adrenoceptors [21-23].

Thus, in the presence of sympathetic nerve fibers in the tissue, norepinephrine would inhibit many proinflammatory activities of neutrophils and monocytes via β_2_-adrenoceptors, which might also play a role in synovial tissue of patients with OA and RA. However, in inflamed synovial tissue of patients with RA, sympathetic nerve fibers are lost and replaced by catecholamine-producing cells [24]. The remaining catecholamine concentrations are low in the synovial tissue, leading to concentrations suitable only for α-adrenergic signaling [24]. Although norepinephrine through α-adrenergic signaling stimulates proinflammatory factors in neutrophils and macrophages [21-23], these low concentrations may well play a proinflammatory role in inflamed tissue.

By using synovial cells of patients with RA, we investigated the effect of norepinephrine on abundance of the proinflammatory bactericidal proteins HNP1-3. Experiments were also carried out in cells from OA patients because possible differences might represent one factor for the differential effects on bone (erosions in RA versus formation of new bone in OA). For comparison, we investigated the human defensin β-defensin 2 (HBD 2). TNF was used as another important proinflammatory stimulus to influence defensin secretion for comparison. In addition, the study aimed to investigate the influence of cortisol alone or together with norepinephrine because cooperative antiinflammatory effects of cortisol and norepinephrine have been described [25].

## Materials and methods

### Patients

In this investigation, we included 10 patients with OA and seven patients with RA. Diagnosis of RA was based on the established criteria according to the American College of Rheumatology (formerly, the American Rheumatism Association) [26]. The characteristics of patients are given in Table 1. Erythrocyte sedimentation rate and serum levels of C-reactive protein were measured by using standard techniques.

**Table 1:** Patient characteristics

	Osteoarthritis	Rheumatoid arthritis
Number	10	7
Age (years)	62.8 ± 3.2	57.0 ± 4.0
Sex (f/m)	6/4	4/3
C-reactive protein (mg/L)	5.1 ± 1.3	25.9 ± 14.1^#^
Erythrocyte sedimentation rate (mm)	11.5 ± 1.5	27.6 ± 3.1*
Lining layer thickness, cells	2.0 ± 0.5	2.5 ± 0.2
Cellular density (all cells), cells/mm^2^	364 ± 169	635 ± 329
T-cell density, cells/mm^2^	1.1 ± 0.1	1.9 ± 0.5
Macrophage density, cells/mm^2^	55 ± 38	52 ± 16
Medication		
Prednisolone (%)	N.A.	86.0
Daily prednisolone (mg/day)	N.A.	6.1 ± 1.7
Methotrexate (%)	N.A.	71.0
Leflunomide (%)	N.A.	14.0
Nonsteroidal antiinflammatory drugs (%)	70.0	71.0

Data are given as mean ± SEM. *P < 0.05; ^#^*P *< 0.005 for the comparison with osteoarthritis. Abbreviations: N.A., not applicable.

The study was approved by the Ethics Committee of the University of Regensburg. Patients were informed about the purpose of the study and gave written consent.

### Synovial tissue preparation, isolation, and culture of primary mixed synovial cells

OA and RA patients underwent elective knee-joint replacement surgery. Synovial tissue samples were obtained immediately after opening the knee-joint capsule, preparation of which was described [24]. In brief, a piece of synovial tissue of ≤9 cm^2 ^was dissected. A larger piece of the synovial tissue was used to isolate mixed synovial cells (for culture experiments, see later). Approximately eight pieces of the same synovial area were used for immunohistochemistry and immunofluorescence, which were fixed for 12 to 24 hours in phosphate-buffered saline (PBS) containing 4% formaldehyde and then incubated in PBS with 20% sucrose for 12 to 24 hours. Thereafter, they were placed in protective freezing medium and quick-frozen (Tissue Tek; Sakura Finetek, Zoeterwoude, The Netherlands). All tissue samples were stored at 80°C.

For culture experiments, mixed synovial cells were isolated during the morning hours by enzymatic digestion for 1 to 2 hours at 37°C by using Liberase (Roche Applied Science, Mannheim, Germany). At approximately 2 to 4 p.m., the synovial cells were resuspended in RPMI 1640 medium (Sigma, Taufkirchen, Germany), supplemented with 1% penicillin/streptomycin (Life Technologies, Inc., Paisley, U.K.) and 0.1% amphotericin B (Bristol-Myers Squibb, Munich, Germany). In total, 2.5 × 10^5 ^isolated synovial cells of OA or RA patients were incubated for 24 hours in the presence of 200 μM L-ascorbic acid, together with norepinephrine, TNF, cortisol, or norepinephrine plus cortisol in indicated concentrations (all substances from Sigma, Steinheim, Germany).

The percentage of different types of synovial cells was tested by specific antibodies against prolyl 4 hydroxylase (for the synoviocyte type B, fibroblasts; Calbiochem, Bad Soden, Germany), CD163 (synoviocyte type A, macrophages; Dako, Hamburg, Germany), CD3 (T cells; Dako), CD19 (B lymphocytes; Dako) neutrophils (elastase; Fitzgerald Industries Int. Inc., Acton, MA, USA), and mast cells (tryptase; Abcam, Cambridge, UK). In preliminary experiments with primary early-culture mixed synoviocytes, we detected that ~37% were positive for prolyl 4 hydroxylase, 26% for CD163, 12% for CD3, 5% for CD19, 10% for elastase, and <1% for tryptase.

### Immunohistochemistry and double immunofluorescence

Approximately ten 5-μm sections were cut from the frozen tissue blocks. For immunohistochemistry with alkaline phosphatase as the enzyme system, sections were blocked with 20% acetic acid for 20 minutes at 4°C. Sections were further blocked with 10% bovine serum albumin, 10% fetal calf serum, and 10% chicken serum for 45 minutes (all from Sigma). Then, sections were incubated with either monoclonal mouse anti-human antibodies against HNP1-3 (BMA Biomedicals, Augst, Switzerland, no. T 1034; dilution, 1:1,000; these antibodies recognize HNP-1 to -3) or polyclonal rabbit anti-human antibodies against HBD-2 (Biologo, Kronshagen, Germany, no. DEF002; dilution, 1:100). Both primary antibodies were incubated overnight at 4°C. After intensive washing with phosphate-buffered saline, sections were incubated with secondary antibodies for 1 hour at room temperature (for HNP1-3, goat anti-mouse coupled to alkaline phosphatase; Dako, no. D0486; dilution, 1:100; for HBD-2, goat anti-rabbit coupled to alkaline phosphatase, Dako, no. D0487; dilution, 1:100). The sections were developed by using BCIP/NBT substrate (Dako, K0598). We controlled specific staining by using irrelevant primary antibodies or serum or by omitting the primary antibody.

Double immunofluorescence was carried out with fixed frozen tissue and a similar blocking procedure to that mentioned earlier. For HNP1-3 and HBD-2, the primary antibodies of BMA Biomedicals and Biologo were used in the same dilution as given earlier. For double staining, respective antibodies were used against activated macrophages (CD163, Dako), T lymphocytes (CD3, Dako), fibroblasts (prolyl-4 hydroxylase, Dako), B lymphocytes (CD19, Dako), mast cells (tryptase; Abcam, Cambridge, UK), and neutrophils (elastase; Fitzgerald Industries International; and Lab Vision NeoMarkers via Thermo Scientific, Dreieich, Germany). Fluorescent staining of positive cells was achieved by incubating the sections with respective secondary Alexa Fluor 488 and 546 antibodies or F(ab')_2 _fragments (Molecular Probes via Invitrogen, Karlsruhe, Germany). Nuclei were stained with Vectashield mounting medium with DAPI (Vector Laboratories via Biozol, Eching, Germany). We controlled specific staining by using irrelevant primary antibodies or serum or by omitting the primary antibody.

### Determination of HNP1-3 and HBD-2 in supernatants of synovial cells

HNP1-3 were measured with commercially available ELISA (HyCult Biotechnology, Uden, The Netherlands; this ELISA recognizes HNP-1 to -3). The detection limit of this assay was 20 pg/ml. Intra- and interassay coefficient of variation were <10%.

With respect to HBD-2, a new ELISA was established by using two commercially available antibodies (capture: Biologo, no. DEF002, polyclonal rabbit anti-human antibodies; dilution, 1:1,000; detection, R&D Systems, Wiesbaden, Germany; AF2758, polyclonal goat anti-human antibodies; dilution, 1:200). After overnight coating with the capture antibody at 4°C, extensive washing, and blocking with 10% fetal calf serum, 100 μl of standard (recombinant HBD-2; Dianova, Hamburg, Germany, no. CYT-26732) or 100 μl supernatant of synovial cells was incubated for 2 hours at room temperature. After extensive washing, the detection antibody was added for another hour (at room temperature). After extensive washing, a rabbit anti-goat tertiary antibody was used, coupled to biotin (Dako, Hamburg, Germany; no. E0466). By using streptavidin coupled to horseradish peroxidase and tetramethylbenzidine (TMB) as the substrate, the ELISA was developed. The detection limit of this assay was ~8 pg/ml. Intra- and interassay coefficients of variation were <15%.

### Superfusion technique of synovial tissue

As described in detail earlier [24], we used a microsuperfusion-chamber apparatus to superfuse pieces of synovial tissue with culture medium. This technique allows the determination of spontaneous defensin release directly from fresh synovial tissue. The superfusion chambers had a volume of ~80 μl. Super-fusion was performed for 6 hours at a tempera-ture of 37°C and a flow rate of 66 μl/min. Synovial tissue pieces had a standard size of 5 μm in diameter with a precision biopsy punch (Stiefel, Offenbach, Germany). Every hour, superfusate was collected to measure HNP1-3 and HBD-2, as described earlier.

### Presentation of the data and statistical analysis

All data are given as mean ± SEM. Box plots give the 10^th^, 75^th^, 50^th ^(median), 25^th^, and 10^th ^percentile. Group medians were compared by using the nonparametric Mann-Whitney test (SPSS/PC, Advanced Statistics, V15.0, SPSS Inc., Chicago, IL, USA). A value of *P *< 0.05 was the significance level.

## Results

### Immunohistochemical localization of HNP1-3 and HBD-2 and double immunofluorescence

**Figure 1:**
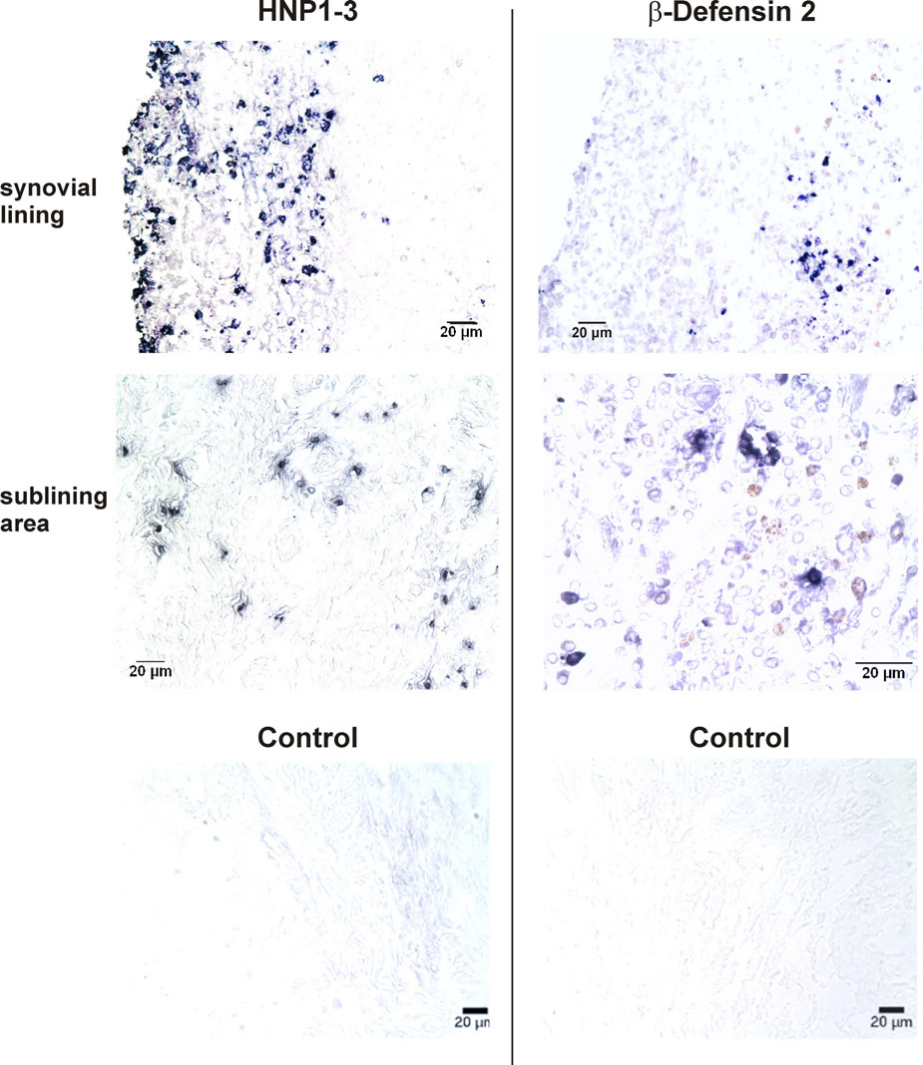
**Immunohistochemistry of human neutrophil peptides 1-3 (HNP1-3) and human β-defensin 2 (HBD 2) in the synovial lining and sublining area of an RA patient (similar in OA patients)**. Antibodies to HNP1-3 stained positive in the lining and adjacent sublining area, whereas HBD-2 was found only in deep sublining zones. Magnification 400×.

To study the localization of defensins, immunohistochemistry of the synovial lining and sublining area was performed in patients with OA and RA. A representative staining of an RA patient is given in Figure 1. HNP1 3 were detected mainly in the synovial lining and in the directly adjacent sublining area (Figure 1, left panels). HNP1-3 were present in most of the RA patients and OA patients. This was quite different for HBD-2, which was rarely detectable in RA and OA patients. The staining in Figure 1, right panels, is a rare example of an RA patient in whom HBD-2-positive cells were detected in the sublining zone.

**Figure 2:**
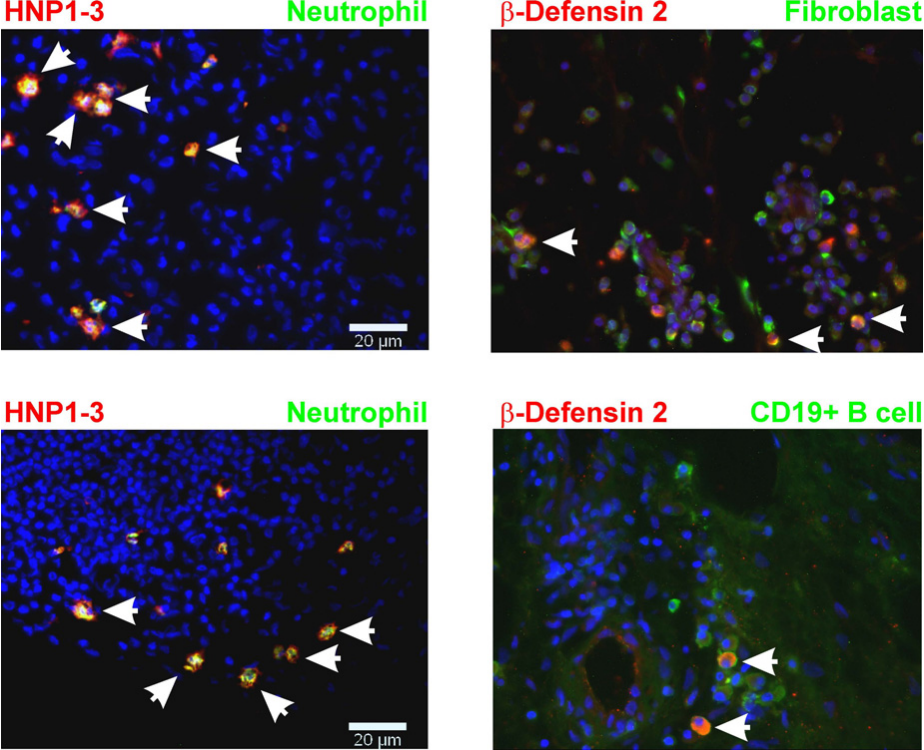
**Double immunofluorescence of human neutrophil peptides 1-3 (HNP1-3) and human β-defensin 2 (HBD 2) in synovial tissue of an RA patient**. Arrowheads show double-positive cells. Left panels: HNP1-3 (red staining) were co-localized with neutrophil elastase (green staining), as indicated by the yellow color in this overlay image. **Right panels**: HBD-2 (red fluorescence) was rarely detected. Some fibroblasts (green fluorescence in the upper right panel) and some CD19^+ ^B lymphocytes (green fluorescence in the lower right panel) stained positive for HBD-2 (yellow overlay). Magnification 400×.

In further extensive double immunofluorescence studies, we provide evidence that HNP1-3 were colocalized to elastase-positive neutrophils (Figure 2, left panels). HNP1-3 were not detected in macrophages, fibroblasts, T lymphocytes, B lymphocytes, or mast cells. Similar to that in immunohistochemistry, HBD-2 was rarely detected with immunofluorescence. In these cases, HBD-2 was colocalized with prolyl-4 of fibroblasts and CD19 of B lymphocytes (Figure 2, right panels). No similar colocalization was observed for macrophages, T lymphocytes, neutrophils, or mast cells.

### HNP1-3 and HBD-2 from synovial tissue and synovial cells

**Figure 3:**
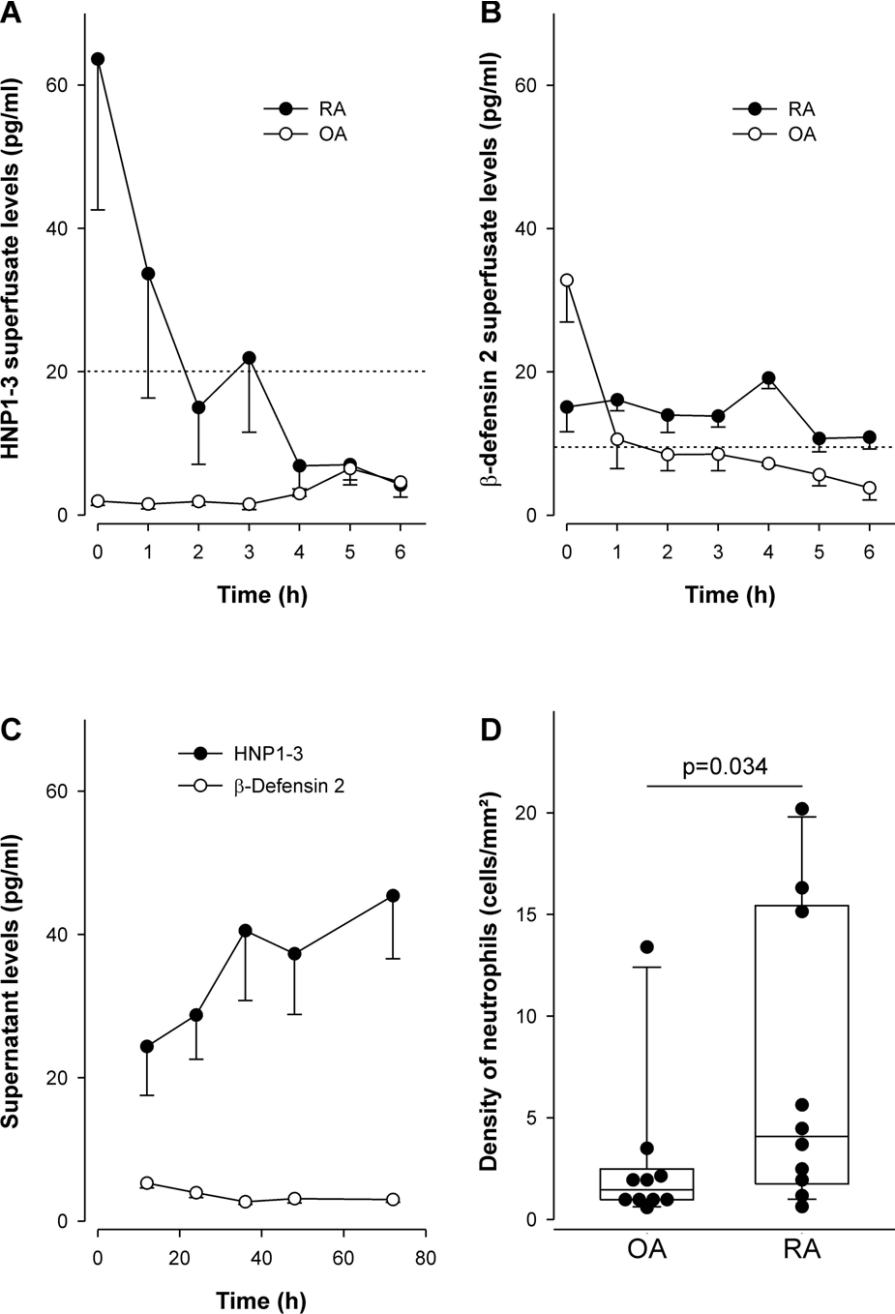
**Secretion of defensins from synovial tissue/cells and density of synovial neutrophils**. **(a) **Levels of human neutrophil peptides 1-3 (HNP1-3) in superfusate of synovial tissue of two patients with rheumatoid arthritis (RA) and osteoarthritis (OA) (four replicates per patient). The dotted line indicates the detection limit. **(b) **Levels of human β-defensin-2 (HBD 2) in supernatant of synovial tissue of two patients with RA and OA (four replicates per patient). The dotted line indicates the detection limit. **(c) **Levels of defensins in supernatant of cultured mixed synovial cells over time. Compare the levels with the detection levels in panels (a) and (b). After 20 hours, HNP1-3 were in the detectable range, which was not the case for HBD-2. The data of four OA patients are given (two replicates of every patient), which was similar in RA patients (data not shown). **(d) **Density of synovial neutrophils in RA and OA synovial tissue. Every symbol represents the average density of neutrophils of one patient, as measured in 17 high-power fields (400×) of two to three synovial tissue sections, including deep sublining areas.

To study the release of HNP1-3 or HBD-2, the protein was detected in the superfusate of synovial tissue. Synovial tissue released HNP1-3, which was particularly evident in RA patients (Figure 3A). The slow decline of superfusate concentration is typical for a washout of the protein and not for a decrease in secretion or production (Figure 3a). Compatible with the findings in immunohistochemistry, HBD-2 superfusate levels were lower, as substantiated in RA and OA patients (Figure 3b).

On a very different time scale, levels of defensins were studied in supernatants of cultured mixed synovial cells (Figure 3c). The levels of HNP1-3 increased over time, whereas HBD-2 was barely detectable (Figure 3c, compare detection limits with those in Figure 3a, b). Because HNP1-3 were detectable by ELISA, further functional studies included only these peptides. We studied early HNP1-3 appearance within 24 hours to minimize a possible effect by necrosis or apoptosis of these cells.

### Density of neutrophils in synovial tissues of patients with OA and RA

The presence of HNP1-3 prompted us to study the density of elastase-positive neutrophils in synovial tissues of patients with OA and RA. The density of neutrophils was higher in RA as compared with OA patients (Figure 3d). The relatively low density of neutrophils is due to the fact that neutrophils were present mainly in the lining and adjacent sublining area but not in deeper layers of the synovial tissue, which were also considered in calculating tissue density (see legend in Figure 3d).

### Influence of TNF, norepinephrine, and cortisol on levels of HNP1-3

**Figure 4:**
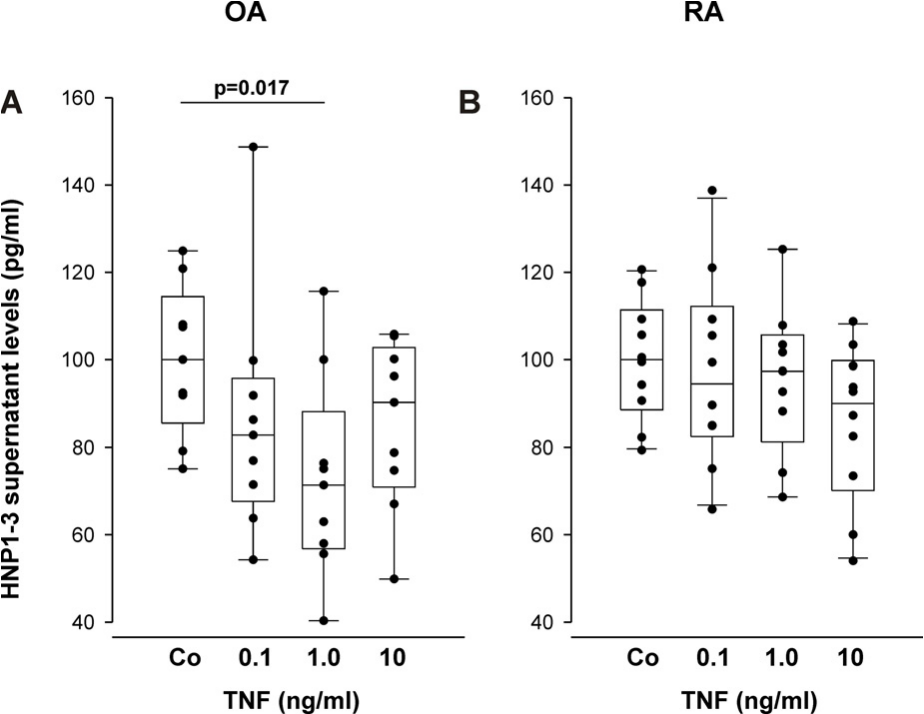
**Influence of tumor necrosis factor (TNF) on levels of human neutrophil peptides 1-3 (HNP1-3) of mixed synoviocytes**. The data are derived from five OA and five RA patients (two replicates of every patient). Control HNP1-3 levels were 58.3 ± 7.0 pg/ml in OA patients and 147.2 ± 37.5 pg/ml in RA patients.

To study important factors that might influence HNP1-3 levels, such as proinflammatory cytokines like TNF, this cytokine was used in culture experiments with mixed synoviocytes. TNF slightly decreased HNP1-3 levels in the supernatant of OA cells, which was significant at 1 ng/ml (Figure 4). In RA synoviocytes, TNF did not exert a similar U-formed dose-response effect because no significant differences were observed (Figure 4).

**Figure 5:**
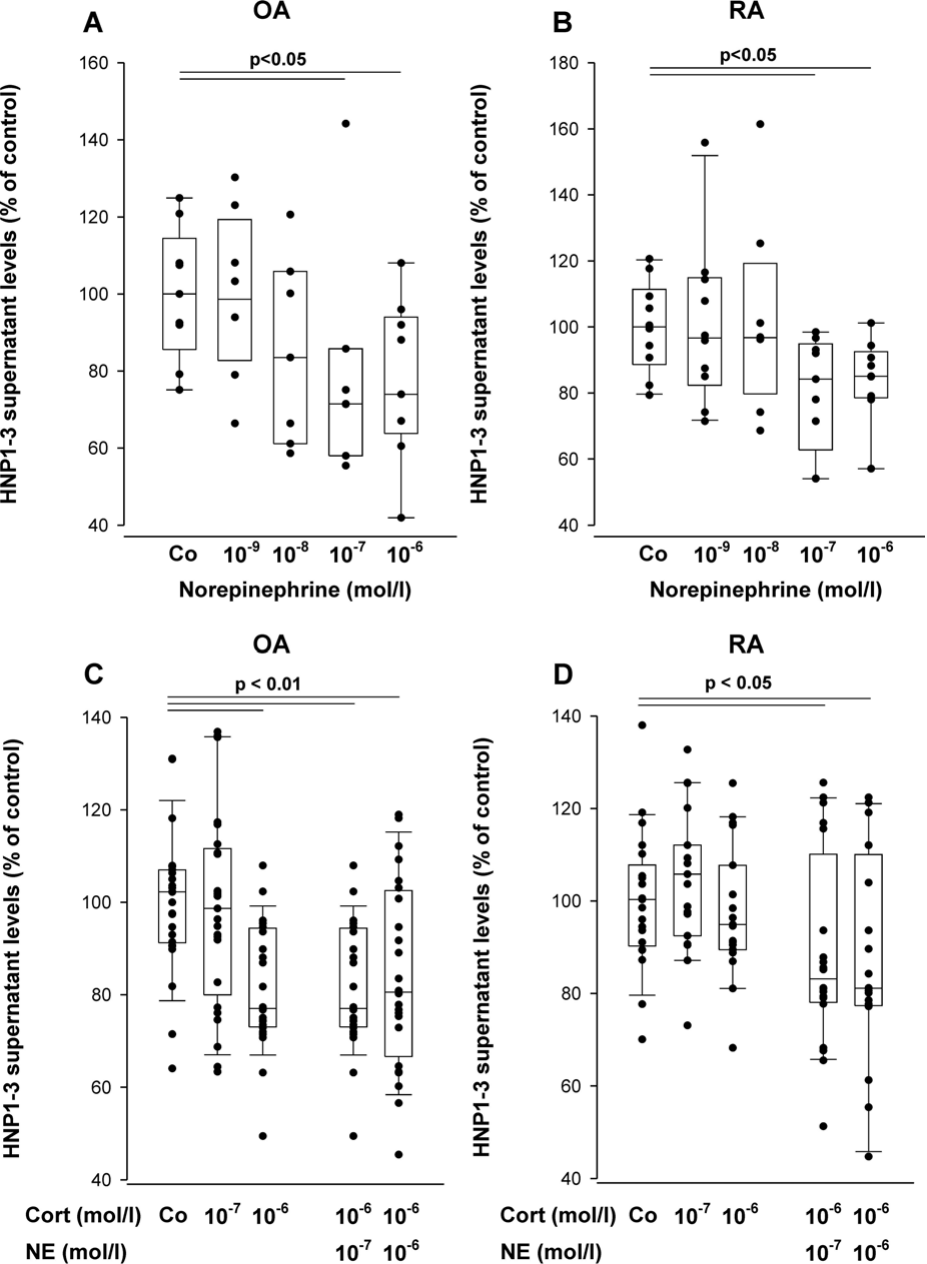
**Influence of norepinephrine (NE) and cortisol (Cort) on human neutrophil peptides 1-3 (HNP1-3) levels in patients with rheumatoid arthritis (RA) and osteoarthritis (OA)**. **(a, b) **Influence of norepinephrine on HNP1-3 levels in supernatants of mixed synoviocytes. The data are derived from five OA and five RA patients (two replicates of every patient). Control HNP1-3 values were as described in the legend of Figure 4. **(c, d) **Influence of cortisol or norepinephrine plus cortisol on HNP1-3 levels in supernatants of mixed synoviocytes. The data are derived from nine OA and seven RA patients (three replicates of every patient). Control HNP1-3 levels were 160.2 ± 50.3 pg/ml in OA patients and 73.5 ± 19.9 pg/ml in RA patients.

In contrast, norepinephrine decreased supernatant HNP1-3 levels produced by mixed synovial tissue cultures in both OA and RA patients (Figure 5a, b). It is obvious that this inhibition was present only at high concentrations when norepinephrine exerts its effects mainly via β-adrenoceptors (Figure 5a, b).

In OA patients, cortisol at high concentrations of 10^-6 ^*M *reduced HNP1-3 concentrations (Figure 5c), which was not observed in RA patients (Figure 5d). In addition, the combination of cortisol plus norepinephrine led to a decrease of HNP1-3 levels produced by mixed synovial tissue cultures in OA and also in RA patients (Figure 5c, d). However, the combined effect of the two hormones did not exceed the individual effects of norepinephrine alone.

## Discussion

This study demonstrates the presence of two human defensins in the synovial tissue of patients with OA and RA. The α-defensin HNP1-3 and the β-defensin HBD-2 were present in synovial tissue, whereas HNP1-3 was undoubtedly allocated to neutrophils (we have not tested for dendritic cells). HBD-2 was found in a small number of fibroblasts and B lymphocytes. Although HNP1-3 were easily detectable by using histologic techniques or in culture experiments, HBD 2 was barely visible in tissue and measurable in supernatants. This study further demonstrated that norepinephrine inhibited HNP1 3 in both RA and OA patients' mixed synovial cell cultures, and cortisol and TNF slightly inhibited this α-defensin only in OA patients. This investigation adds to the understanding of how the SNS might influence HNP1-3 in chronic inflammatory joint diseases.

In a previous report, the presence of HNP1 3-positive cells was demonstrated in the synovial lining of healthy subjects and in patients with suppurative arthritis, OA, and RA [8]. However, these authors did not study the regulation of HNP1-3, which was a particular aspect in the present work. Another study reported very high synovial fluid levels of HNP1 3 in RA patients, associated with more-severe erosions [9]. Because we used the same ELISA mentioned in the latter study, we were surprised that our superfusate and supernatant levels of HNP1-3 were lower. We interpret these earlier findings of Bokareva *et al. *[9] insofar as HNP1-3 accumulate in the synovial fluid, which has been demonstrated for other factors as well (for example, estrogens [27]). Because we have not studied synovial fluid levels, we cannot directly compare the results of the two studies. However, discrepant findings might also depend on increased abundance of neutrophils in synovial fluid, as compared with synovial tissue.

Although HNP1-3 defensins have been reported to be expressed by granulocytes in synovial tissue [8], it was not clear whether these proteins are actually released in the tissue. By using the superfusion approach, we were able to demonstrate that these proteins are released by few granulocytes in synovial tissue. In the present study, HNP1-3 is produced in higher amounts in RA than in OA, which might reflect the overall greater inflammation in patients with chronic RA. This is particularly relevant because RA patients were treated with prednisolone and antiproliferative drugs that should have reduced proinflammatory factors like HNP1-3. HNP1-3-positive cells were elastase-positive neutrophils, and we can exclude that other cells such as macrophages, fibroblasts, T and B lymphocytes, or mast cells stain positive for this protein. However, we have not tested dendritic cells that can also produce HNP1-3 [10,11]. The relatively low number of neutrophils in the tissue might be a consequence of rapid migration into the synovial fluid (>95% are neutrophils).

The mentioned first study of Paulsen *et al. *also reported on HBD-2 [8], which was not found in their samples investigated (they found the defensins HBD-3, CAP37, and LL37). We confirmed that immunohistologic detection of HBD-2 is a rare event (found in only one patient with RA and in one with OA). Nevertheless, this RA patient had numerous positive cells that allowed double staining, revealing double-positive fibroblasts and B lympho-cytes. Because HBD-2 levels were below the detection limit in most superfusate and supernatant samples, functional experiments with TNF and hormones aimed only to investigate the α-defensin HNP1-3. Further studies corroborated that neutrophils are present in OA and RA tissue, and that the density of these cells is higher in RA than in OA patients. It is known that the SNS has a strong influence on neutrophils (but also on monocytes), so we were particularly interested in the functional effects of the SNS on HNP1-3.

The major neurotransmitter of sympathetic nerve fibers, norepinephrine, inhibits several cellular functions. For example, norepinephrine decreases migration [17,18], oxygen radical production [19], phagocytosis [18], and bactericidal activity [20]. These inhibitory influences on neutrophils (but also on monocytes) were present only when norepinephrine appeared at high concentrations (via β_2_-adrenergic receptors). This might be quite different at low concentrations, when norepinephrine exerts its effects via α-adrenoceptors [21-23]. Our present study supports the inhibitory influence of norepinephrine, focusing on the α-defensin HNP1-3. In both RA and OA patients, norepinephrine dose-dependently decreased supernatant levels of HNP1-3 from mixed synovial tissue cultures, which reached the significance level only at high concentrations. These norepinephrine effects were stronger when compared with cortisol alone, which did not influence HNP1-3 in RA synovial cells. In addition, the combination of norepinephrine plus cortisol did not materialize in an additive or even synergistic effect. These results confirm the overall inhibitory effect of norepinephrine on cellular functions, given that concentrations of this neurotransmitter are high enough. At low concentrations of 10^-9 ^*M *norepinephrine, we did not observe opposite effects that would indicate a proinflammatory influence on mixed synovial tissue cultures via α-adrenoceptors.

Because TNF is an important proinflammatory molecule in chronic inflammatory joint diseases, the effect of this cytokine on HNP1-3 levels was tested in this study. It was known that β-defensins such as HBD-2 can be stimulated by TNF in epithelial cells and astrocytes [28,29], but, to our knowledge, effects of TNF on HNP1-3 were not investigated (especially not in OA/RA material). TNF decreased HNP1-3 supernatant levels in OA mixed synovial cultures, and the dose-response curve was U-shaped with a maximum inhibition at 1.0 ng TNF/ml. TNF did not exert similar effects in RA cell cultures, which may be related to already high TNF levels in these cell preparations (receptor desensitization). Because, conversely, HNP1-3 can stimulate TNF secretion from macrophages, as recently reported [7], the TNF-induced inhibition of HNP1-3 might be part of a negative-feedback loop that prevents overshooting of innate immune responses. From this point of view, we hypothesize that HNP1-3 precedes TNF in the stimulatory cascade of innate immunity events. This would be an important aspect because TNF has been installed at the forefront of the innate immunity cascade in RA [30].

## Conclusions

In conclusion, HNP1-3 is present in neutrophils, and as compared with HBD-2, it is abundantly present in chronically inflamed synovial tissue of patients with RA and OA. Because HNP1-3 levels are decreased by norepinephrine in mixed synovial-cell cultures, this study demonstrates that the SNS might inhibit neutrophil (but also monocyte) function *in vivo*. Sympathetic nerve fibers are lost in inflamed tissue [24,31], so concentrations of this neurotransmitter are probably too low to induce antiinflammatory activities. A similar situation exists for TNF, which is also decreased by norepinephrine [32]. HNP1-3 can stimulate secretion of TNF [7], so the loss of sympathetic nerve fibers with low concentrations of norepinephrine might have double-negative effects, possibly increasing both local HNP1-3 and TNF levels. This scenario can be relevant in overt inflammation of the tissue when monocytes (HNP1-3, TNF), macrophages (TNF), and neutrophils (HNP1-3) play a decisive role.

## Competing interests

The authors declare that they have no competing interests.

## Authors' contributions

BR and SG participated in the concept and design and acquisition of data. RW and MF participated in interpretation of data and in drafting and revising the article. RHS participated in the concept and design, analysis and interpretation of data, and drafting and revising the article.
